# Morphological evolution and land cover changes in the Mureș River riparian area over a century of anthropogenic pressure

**DOI:** 10.1007/s10661-025-14251-8

**Published:** 2025-06-24

**Authors:** Mihai Daniel Niță, Jorge Diaz Suarez, Ioana Alexandra Nicolae

**Affiliations:** Department of Forest Engineering, Faculty of Silviculture and Forest Engineering, Transilvania University of Brasov, Brasov City, Romania

**Keywords:** River morphology, Riparian Zone, Land cover change, Geomatic techniques, Urbanization, Geomorphology

## Abstract

This study provides a comprehensive analysis of the morphological changes in the Mureș River channel and the associated land cover changes within the riparian zone over a century, from 1900 to 2005. Utilizing advanced geomatic techniques, the research reveals significant alterations in the river’s geometry and riparian land cover, influenced by both natural dynamics and human activities. Our findings reveal a significant decrease of approximately 7.5% in the river’s surface area, indicating substantial geomorphological transformations. Meander characteristics exhibited notable alterations: some sectors experienced an increase in meandering by up to 60%, reflecting natural adjustments to environmental conditions, while others showed significant straightening with decreases in meander length by up to 50%, likely due to human interventions such as river engineering and land use changes. The land cover analysis within the riparian zone highlighted a substantial increase of 257% in urban construction, expanding from 5060 hectares in 1900 to 13,045 hectares in 2005. Concurrently, there was a 16% reduction in forested areas, with a loss of 4234 hectares, reflecting intense urbanization and deforestation trends. These changes were more pronounced in sectors adjacent to urban centers, indicating the significant impact of human activities on the river’s environment. The findings show a notable decrease in the river’s surface area and an increase in meandering in certain sectors, while others have experienced significant straightening. The land cover analysis highlights a marked increase in urban construction and a significant reduction in forested areas, reflecting broader trends of urbanization and environmental transformation. These insights are crucial for informing future river management and conservation strategies, emphasizing the interconnectedness of geomorphological processes and land use practices.

## Introduction

Rivers are dynamic natural systems that continuously reshape the landscapes they traverse, influencing ecological processes and supporting human societies (Germaine & Gonin, 2024; Reviews 2015). Over time, river channels undergo morphological changes due to natural dynamics such as erosion, sediment transport, and deposition (Hamidifar et al., 2024). These processes are often compounded by anthropogenic activities, including urban development, agricultural practices, and industrial operations, leading to significant alterations in river morphology and adjacent land cover (Khatri & Tyagi, 2015; Liu et al., 2023). The river’s morphology, encompassing the shape, size, and path of its channel, plays a critical role in determining the hydrological responses of the river system (Walling, 2006). The geometry of the channel directly affects the river’s flow kinetics, shaping the movement of water and its interaction with the riverbed and banks (Dong et al., 2024). Understanding these changes is essential for effective river management, conservation strategies, and sustainable development.

The Mureș River, the second-longest river in Romania, is a vital component of the environmental and economic landscape of Transylvania. Originating from the Eastern Carpathians in the Hășmașu Mare Range, it flows westward for approximately 761 km before joining the Tisa River (Vigh & Csaba, 2024). The river traverses a diverse range of landscapes—including alpine regions, forests, agricultural lands, and urban areas—each exerting significant influence on its hydrology and morphology. This diversity makes the Mureș River an ideal subject for examining the long-term impacts of environmental changes and human interventions on river morphology.

Despite the significance of the Mureș River, there is a notable lack of comprehensive, long-term studies that integrate morphological changes and land cover dynamics within its basin (Peptenatu et al., 2020). Previous research has often been limited to short-term observations or focused on isolated aspects of river dynamics without considering the combined effects of natural processes and anthropogenic pressures over extended periods (Vigh & Csaba, 2024). For instance, studies may have examined sediment transport or local erosion patterns without linking these to broader land use changes or historical modifications of the river channel (Jurcoane et al., 2023). This limitation hinders a holistic understanding of the river’s evolution and the underlying mechanisms driving these changes (Morar et al., 2020).

Our study addresses this gap by providing a century-long analysis of the Mureș River’s morphological evolution and associated land cover changes within its riparian zone from 1900 to 2005. By utilizing advanced geomatic techniques—including Geographic Information Systems (GIS), remote sensing, and historical cartographic analysis—we offer a unique perspective on the river’s evolution that previous studies have not captured. This long-term approach allows us to observe patterns and trends not apparent in short-term studies, thereby enhancing our understanding of the river’s response to both natural dynamics and human activities (Nita et al., 2018). These patterns, although simple, underscore the significant interaction between hydraulic and geometric parameters within a river’s alluvial bed (Gao & Li, 2024). The concept of morphometric relationships is based on the principle of minimal energy dissipation or maximum discharge efficiency (Richards, 2024). However, these relationships remain largely empirical, relying heavily on direct data processing from observations and measurements without a comprehensive theoretical framework to fully explain the phenomena of channel formation in alluvial rivers (Gao & Li, 2024). This gap highlights the need for continued research to develop more robust theoretical models.

A key aspect of our research is exploring the relationship between land cover change and river morphology in the Mureș River. Land use practices such as deforestation, urbanization, and agricultural expansion within the riparian zone can significantly alter runoff patterns, sediment loads, and bank stability. These changes can lead to modifications in channel geometry, meandering patterns, and flow dynamics. By analyzing land cover changes alongside morphological data, we can assess how these anthropogenic activities have influenced the river’s natural processes. Understanding these underlying mechanisms is crucial for interpreting the morphological changes observed in the Mureș River and for predicting future alterations.

Meandering occurs as the river erodes its banks and deposits sediments on the inside of bends, gradually shifting its channel across the floodplain (Finotello et al., 2024). This dynamic is influenced by the river’s load of suspended sediments and flow energy, which together dictate the extent and form of meandering (Gao & Li, 2024). The basic morphometric unit of a meander includes its wavelength (λ), the distance between the points of maximum curvature of successive bends, and its amplitude, collectively describing the geometry of the river’s path. Understanding these elements is crucial for predicting future changes in the river’s course and for planning effective river management strategies.

Research on river dynamics increasingly emphasizes the need for a comprehensive approach that considers both the physical processes of sediment transport and deposition, as well as the ecological aspects of river corridors (Krishnan et al., 2024). Rivers are not isolated entities but are integrally connected to their landscapes (El Jeitany et al., 2024). Changes in land use, climate, and hydrological regimes significantly impact river morphology and ecosystem services (El Jeitany et al., 2024; Rahimi et al., 2024). The Mureș River is no exception, with its floodplain and associated ecosystems undergoing notable changes due to alterations in river flow and sediment load driven by both natural variability and human-induced changes (Jurcoane et al., 2023; Morar et al., 2020; Peptenatu et al., 2020).

The river’s floodplain is particularly sensitive to changes in land use and hydrological regimes. Urbanization and agricultural expansion can lead to increased surface runoff and sediment load, altering the natural sediment transport processes and affecting the river’s morphology (Liébault et al., 2005). Additionally, climate change poses a significant threat to the stability of river systems worldwide, including the Mureș River. Changes in precipitation patterns and temperature can influence the frequency and intensity of flooding events, further complicating the management of river basins (Krishnan et al., 2024).

Geomatic techniques, including Geographic Information Systems (GIS), remote sensing, and historical cartographic analysis, have emerged as essential tools for studying river dynamics. These techniques provide a unique vantage point to observe and quantify changes over extended periods, offering critical data for effective river basin management and environmental conservation (Chatrabhuj et al., 2024). The strength of these techniques lies in their ability to integrate diverse data sources into a coherent spatial and temporal analysis framework, revealing patterns not apparent through traditional observational methods alone (Escandón-Panchana et al., 2024; Rosa et al., 2024).

We employ a combination of historical maps and modern GIS tools to analyze morphological evolution and spatiotemporal land cover changes. The use of historical cartographic data, such as the maps from 1900, provides a detailed portrayal of the river’s past state. By digitizing and georeferencing these maps, we can compare historical river geometries with contemporary data derived from satellite imagery and updated GIS datasets. This methodological approach allows for precise quantification of changes in river morphology and land cover over a century, setting our study apart from others that may lack such temporal depth or spatial accuracy.

The findings of our study have significant implications for river management and conservation strategies in the Mureș River basin and beyond. By highlighting the extent of morphological changes and land cover transformations over a century, we emphasize the need for integrated approaches that consider both geomorphological processes and land use practices. Our research can inform policymakers, environmental managers, and stakeholders about the impacts of human activities on river systems, aiding in the development of sustainable management practices that balance ecological integrity with economic development. Furthermore, the methodologies and insights from our study can be applied to other river systems facing similar challenges, contributing to global efforts in river conservation and sustainable land use planning.

This study aims to investigate the morphological evolution and land cover changes in the Mureș River basin over a century, by addressing three objectives. The first objective is to quantify morphological changes in the riverbed geometry. This involves measuring meander characteristics such as wavelength and amplitude to understand the degree of meandering and the river’s dynamic equilibrium state. The second objective is to analyze land cover changes within the riparian zone. We perform land cover classification using both historical and contemporary aerial imagery to categorize land cover types—such as forested areas, agricultural land, urban development, and natural vegetation. The third objective is to assess the relationship between land cover changes and river morphology. We examine how alterations in riparian land use influence river dynamics by correlating changes in land cover with modifications in the river’s geometry and flow patterns. Employing statistical tools, we aim to provide insights into how land use impacts river health and behavior. This includes analyzing the influence of riparian land cover changes on meandering patterns, channel geometry, and hydrological responses.

## Methods

### Study area

**Fig. 1:**
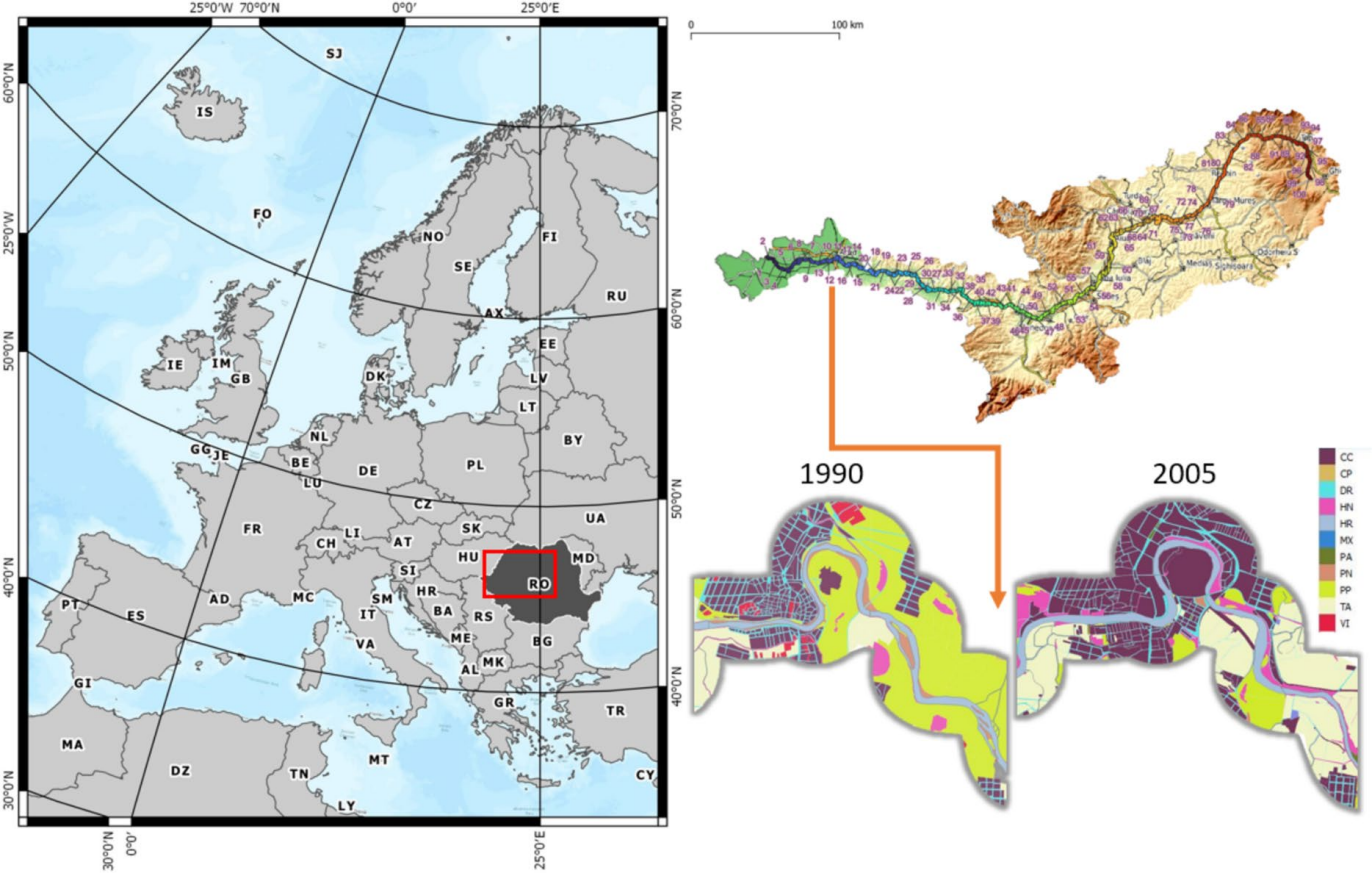
Study area of Mures Basin

The Mureș River is a significant river system located entirely within Romania, flowing westward from its source in the Eastern Carpathians through the historical region of Transylvania before joining the Tisa River, a tributary of the Danube (Fig. 1). Spanning approximately 761 km, it is the second-longest river in Romania. The river’s basin covers an area of about 27,890 square kilometers and encompasses several counties, including Harghita, Mureș, Alba, Hunedoara, and Arad.

The river traverses a diverse range of landscapes. In its upper reaches, the Mureș flows through mountainous terrains characterized by steep slopes, narrow valleys, and coniferous forests. Mid-sections of the river are flanked by mixed forests and agricultural lands, providing vital ecological habitats and supporting local economies. As it descends into lower elevations, the river passes through extensive agricultural zones where crops such as wheat, maize, and sunflowers are cultivated. The Mureș also flows through several urban areas, including the cities of Târgu Mureș, Alba Iulia, and Arad, contributing to the socio-economic development of the region.

### Data

For this study, a dataset was compiled to provide both spatial and temporal coverage of the Mureș River’s morphological characteristics from 1900 to 2005.

For the 1900 period as historical map, a specific source of topographic maps dating back was used, “Plan Director de Tragere” maps, 1:20,000 scale. These maps, numbering 2118 plan sheets, provided a detailed portrayal of Romania’s topography at the time. Each sheet, measuring 75 cm in length and 50 cm in width, translated to 15 km and 10 km on the ground, respectively, offering a meticulous view of the country’s landscapes. For Mures watershed, the maps were derived from Austrian topographic maps. These maps were based on the Lambert-Cholesky projection system georeferenced during e-Harta project and were reprojected in EPSG:3844 on the fly using QGIS.

For contemporary data, GIS datasets were obtained by digitizing and updating TOPRO5 dataset based on the aerial ortophotomaps. These datasets included digital elevation models (DEMs), land use maps, and hydrological data of the river basin. Recent satellite imagery was also utilized to assess current conditions and validate historical map data. Spatial analyses were performed to identify and quantify changes in the river’s morphology and adjacent land uses. The integration of these data sets allowed for a multi-temporal analysis that tracks the evolution of the riverbed’s geometry over the last century as well as the land use changes.

### Measurement of river morphology

Understanding the river’s geomorphology involves detailed measurement of its channel, which includes the riverbed’s shape, size, and course. These features significantly influence the river’s flow dynamics and are subject to changes over time due to natural processes and anthropogenic activities. The morphology of river channels is dictated by a dynamic balance between the sediment load carried by the river and the landscape through which it flows. This balance impacts erosion, sediment transport, and deposition processes, which in turn affect the river’s channel formation and alteration.

**Fig. 2:**
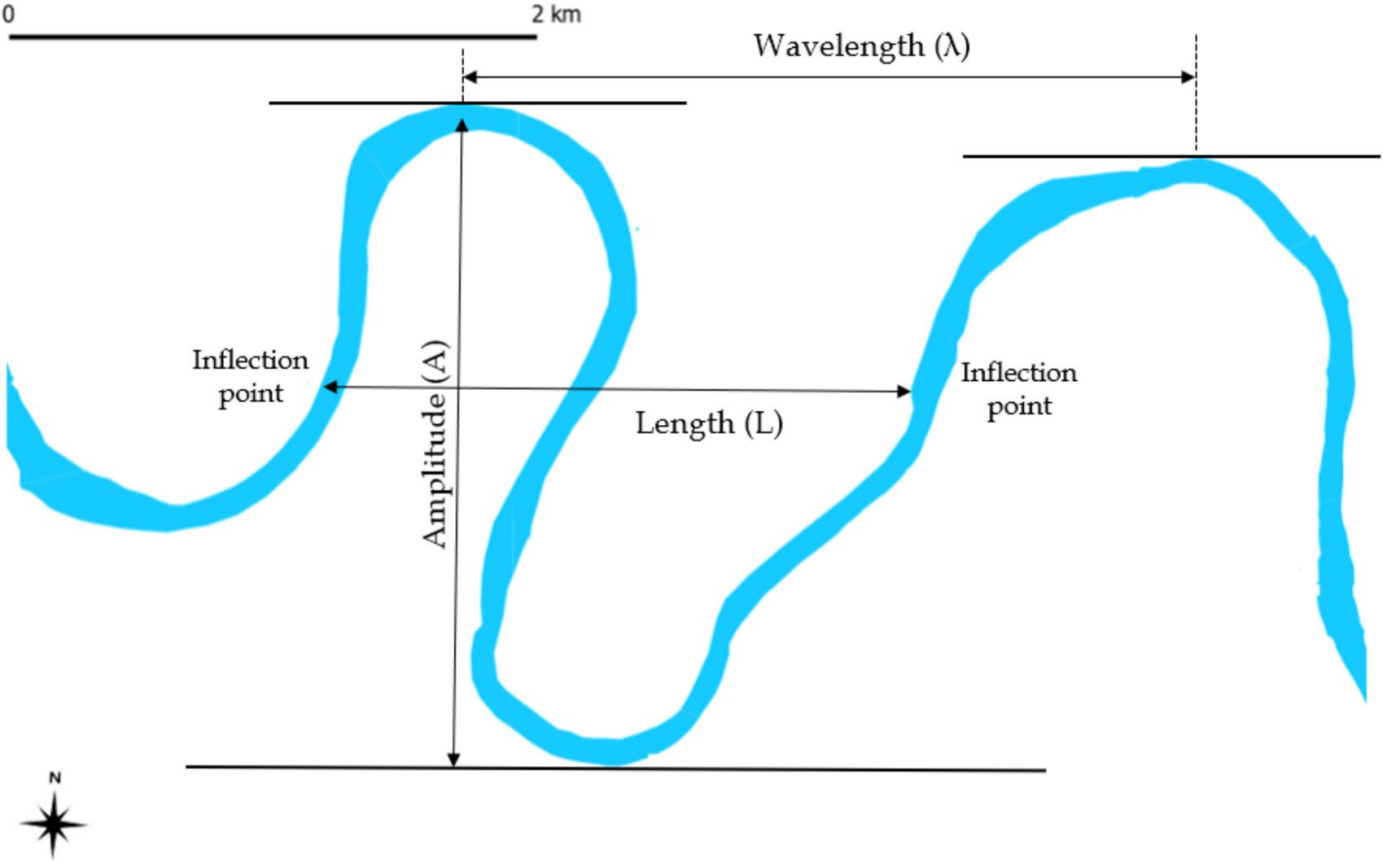
Meander amplitude, length, and wavelength

The morphometric analysis of the Mureș River involved several detailed steps to understand its dynamic changes over time. The first step was measuring the river meanders, where the study quantified characteristics such as wavelength (*λ*), which is the distance between points of maximum curvature of successive bends, and amplitude (Fig. 2). These measurements are critical for understanding the degree of meandering, which indicates the dynamic equilibrium state of the river.

Next, the analysis focused on channel changes by employing techniques like cross-sectional analysis at multiple points along the river. This approach allowed for the assessment of changes in the width, depth, and sinuosity of the river over time. Understanding these changes is essential for grasping how the river’s physical structure evolves in response to various factors.

Empirical data processing was another vital step, involving the collection of field observations and GIS analyses. This data was processed to establish correlations between the river’s physical changes and the geomorphological processes driving these changes. Traditionally reliant on direct observation, this empirical approach has been significantly enhanced by modern computational methods, providing a more detailed and comprehensive understanding of river dynamics.

Integration of historical and modern data was achieved using QGIS to create attribute tables detailing the river’s geomorphological states over time. These tables were exported to Microsoft Excel for further analysis, allowing researchers to evaluate trends in the river’s dynamic changes and their implications for river management.

### Analysis of land cover changes in the riparian zone

To understand the ecological impacts and land use dynamics within the riparian zone, we conducted a detailed land cover change analysis. Land cover was classified into forested areas, agricultural land, urban development (constructed areas), natural vegetation, and water bodies.

For the historical land cover (1900), features were digitized from the georeferenced “Plan Director de Tragere” maps. Map symbology and annotations were used to interpret land cover types, and historical records were consulted to resolve any ambiguities. For contemporary land cover (2005), high-resolution satellite imagery and aerial photographs were utilized. Supervised classification was performed using the “Semi-Automatic Classification Plugin” in QGIS. Representative samples for each land cover class were selected to generate spectral signatures, and the classification results were verified through field visits where possible.

Change detection was conducted by comparing the classified land cover maps from 1900 and 2005. A post-classification comparison method was employed, where the two land cover maps were overlaid, and changes between classes were identified on a pixel-by-pixel basis. A land cover transition matrix was created to quantify the changes between classes over the study period. GIS overlay analysis, using the “Intersect” tool in QGIS, was applied to produce a change map highlighting areas where land cover types transitioned from one class to another. The extent of land cover changes was quantified by calculating the area of each class and the changes between classes using the “Calculate Geometry” function, and changes were expressed in both absolute terms (hectares) and percentages.

An accuracy assessment was performed to evaluate the reliability of the land cover classifications. For the historical data, validation was challenging due to the inability to conduct ground truthing; however, accuracy was assessed by cross-referencing with historical documents and literature.

### Statistical analysis

To explore the relationship between land cover changes and river morphology, statistical analyses were performed. Data on morphological parameters and land cover changes were compiled into attribute tables in QGIS and exported to statistical software (R) for analysis. Ordinary least squares (OLS) regression was used to assess linear relationships between changes in river morphology (dependent variables) and changes in land cover types within the riparian zone (independent variables).

The regression models included changes in meander amplitude, wavelength, and sinuosity as dependent variables and percentage changes in forest cover, agricultural land, and urban development as independent variables. Variance inflation factor (VIF) was calculated to check for multicollinearity among independent variables. Statistical significance was determined using *p* values and confidence intervals, and *R*-squared values provided the proportion of variance in the dependent variable explained by the model.

Spatial autocorrelation in the residuals of the regression models was assessed using Moran’s *I* statistic. If significant spatial autocorrelation was detected, spatial regression models, such as the spatial lag model, were considered to account for spatial dependencies in the data. Finally, the relationship between land cover changes and river morphology was examined to understand the influence of riparian land use on river dynamics. Statistical tools were employed to correlate changes in land cover with alterations in the river’s geometry and flow patterns, providing insights into how land use impacts river health and behavior.

## Results

The analysis of the Mureș River in one century revealed significant alterations in both its channel geometry and the adjacent riparian land cover. By integrating historical maps with contemporary GIS data, we quantified these changes and assessed their spatial and temporal variations across different river sectors.

### Changes in the riverbed morphology

**Fig. 3:**
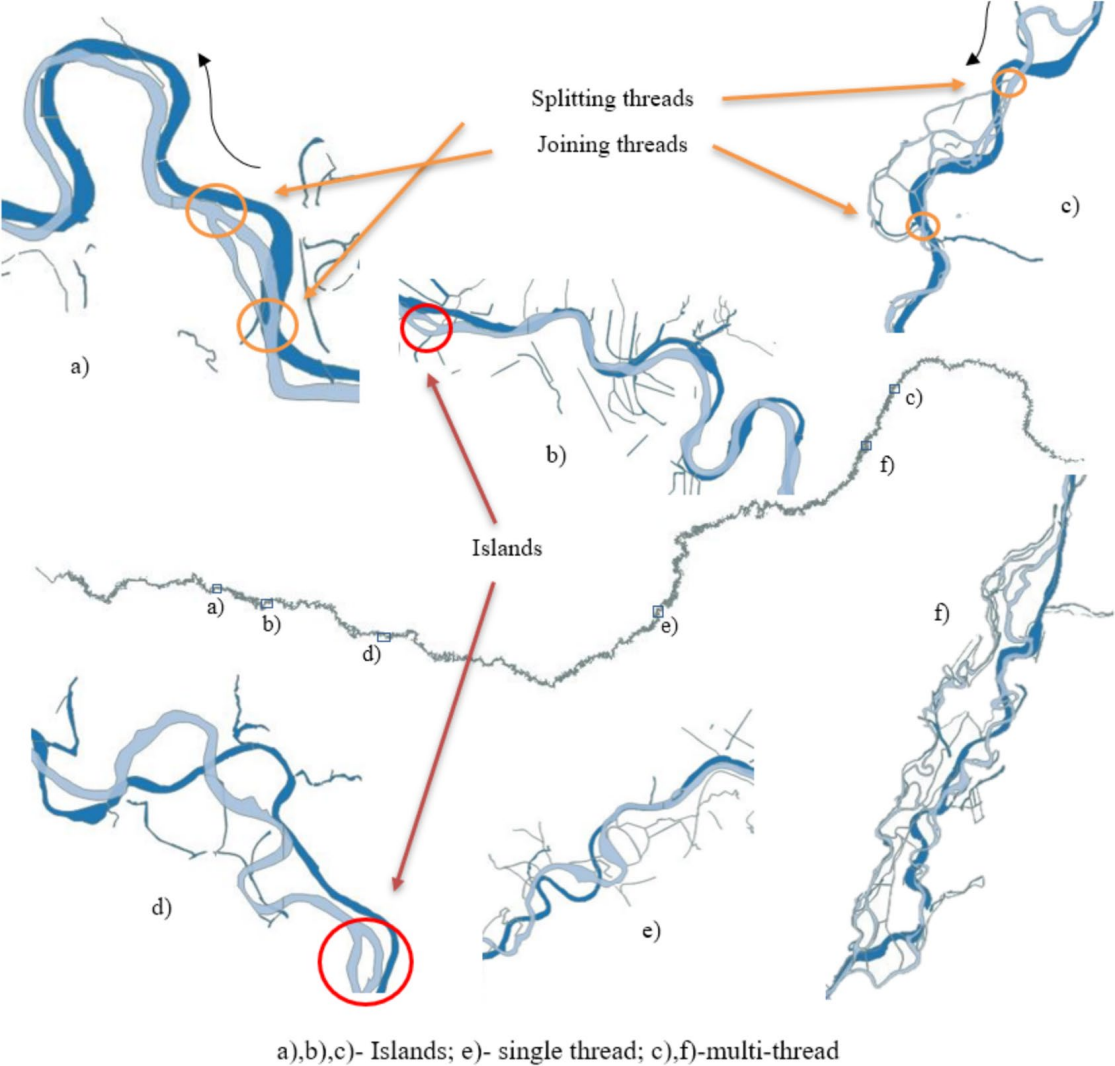
Visual examples with changes in Mures riverbed

The Mureș River exhibited notable changes in its channel geometry over the observed century. Overall, there was a discernible decrease in the river’s surface area, along with alterations in meander patterns and channel dimensions (Fig. 3).

**Fig. 4:**
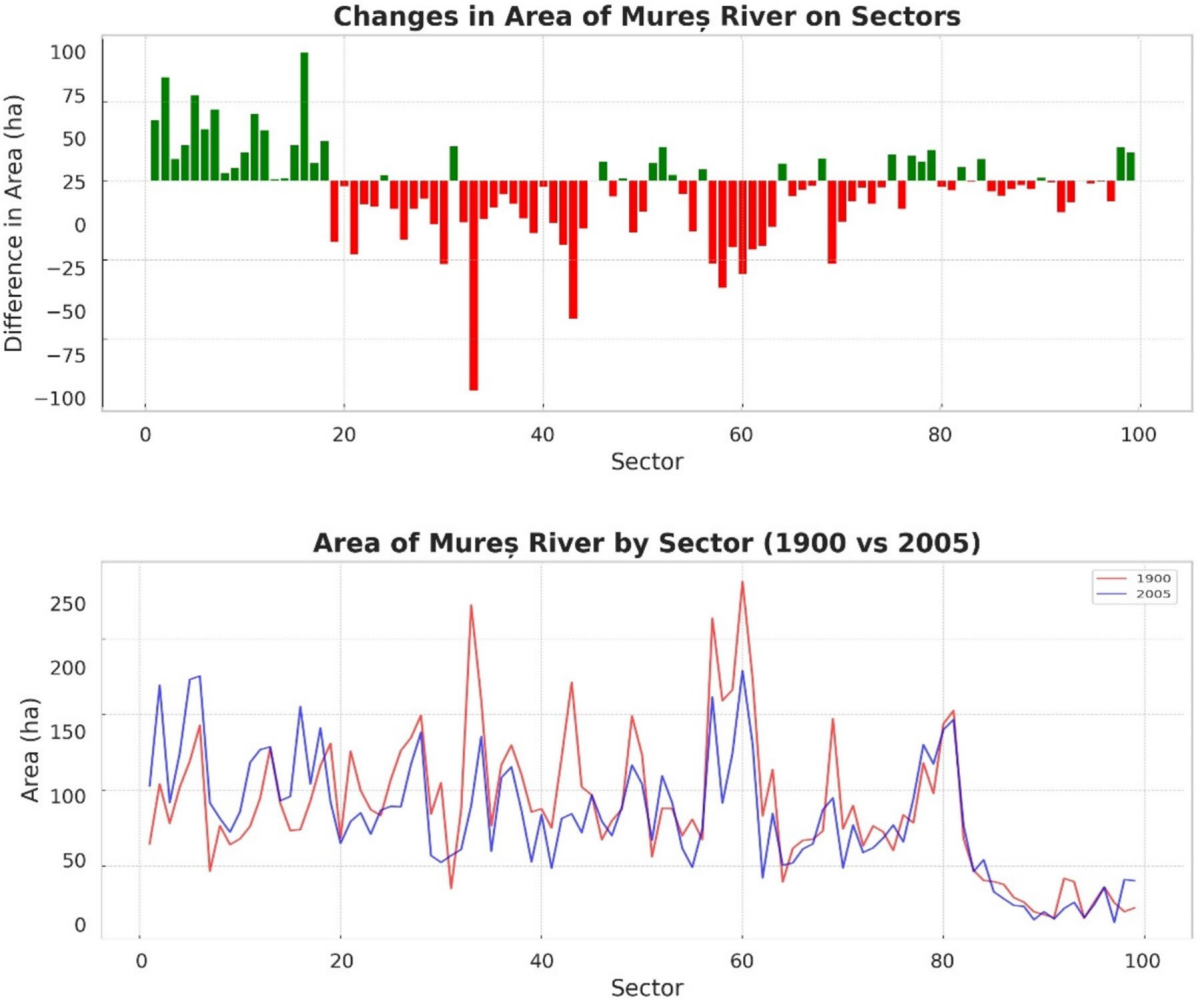
Changes in the riverbed areas in percentage and area (ha) distribution

From the point of view of the riverbed, we observe that although in the first 20 sectors the riverbed has shown an increase since 1900, overall, it shows a negative change. However, the calculations show that the whole area of the Mures River in 2005 has decreased by about 7–8% compared to 1900, specifically from 8845.62 to 8175.83 ha. This trend varied significantly depending on the geographical terrain—plain areas showed higher susceptibility to erosion, affecting river dynamics differently than in hilly or mountainous areas (Fig. 4).

Key findings from the study of the Mureș River channel geometry include several points of interest. The meander characteristics such as wavelength (*λ*), amplitude, and the distance between points of maximum curvature have varied over time, reflecting a shift in the river’s equilibrium state. Notably, in sector 40, there was an observed increase in amplitude by 7.40% from 1900 to 2005, suggesting an intensification of the meandering process.

Regarding cross-sectional changes, the river’s width, depth, and sinuosity have both increased and decreased in different sectors. There was a general decrease in the river surface area by approximately 7–8%, with a reduction from 8845.62 hectares in 1900 to 8175.83 hectares in 2005, indicating significant alterations in the riverbed’s dimensions over the century.

**Fig. 5:**
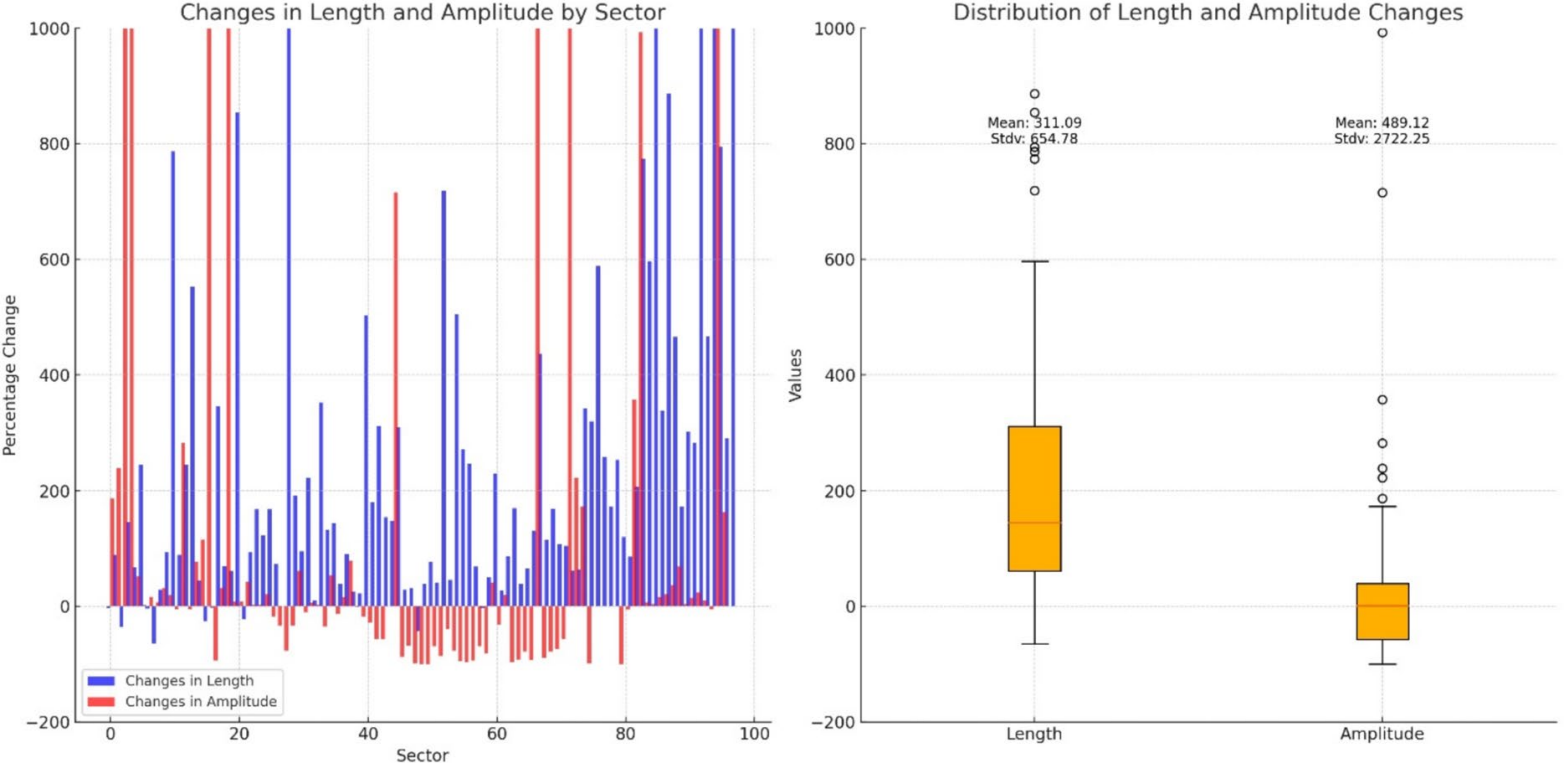
Changes in length and amplitude over a century in Mures sectors

The dynamics of the river’s channel was also evident in the meandering coefficient, which experienced significant changes, particularly noted between sectors 43 and 46. This points to variations in the river’s sediment transport and deposition patterns, affirming the dynamic nature of the river’s morphology. The study revealed that compared to the year 1900, meanders have altered their lengths and amplitudes both positively and negatively, showcasing the river’s ongoing response to various influencing factors (Fig. 5).

Regarding the variability in Meander Geometry, there is considerable variability across the sectors. Some sectors, like 42 and 69, experienced a significant increase in length, 49% and 60% respectively, suggesting that the river has become more sinuous in these areas. Conversely, other sectors like 43 and 44 saw decreases in length by 30% and 50%, indicating a straightening of the river course there.

The amplitude of meanders also highly changed in several sectors. Notable increases in amplitude are seen in sector 51 with a 50% increase, implying a widening of the meander bends. However, many sectors such as 46 and 58 show substantial decreases in amplitude, up to 60% and 22%, respectively, which may reflect river engineering efforts or natural adjustments in the river’s course.

Sectors 40 to 46 seem to display the most significant changes, with many of these sectors showing a decrease in both length and amplitude, suggesting considerable alterations in the river channel.

### Changes in the riparian zone

**Fig. 6:**
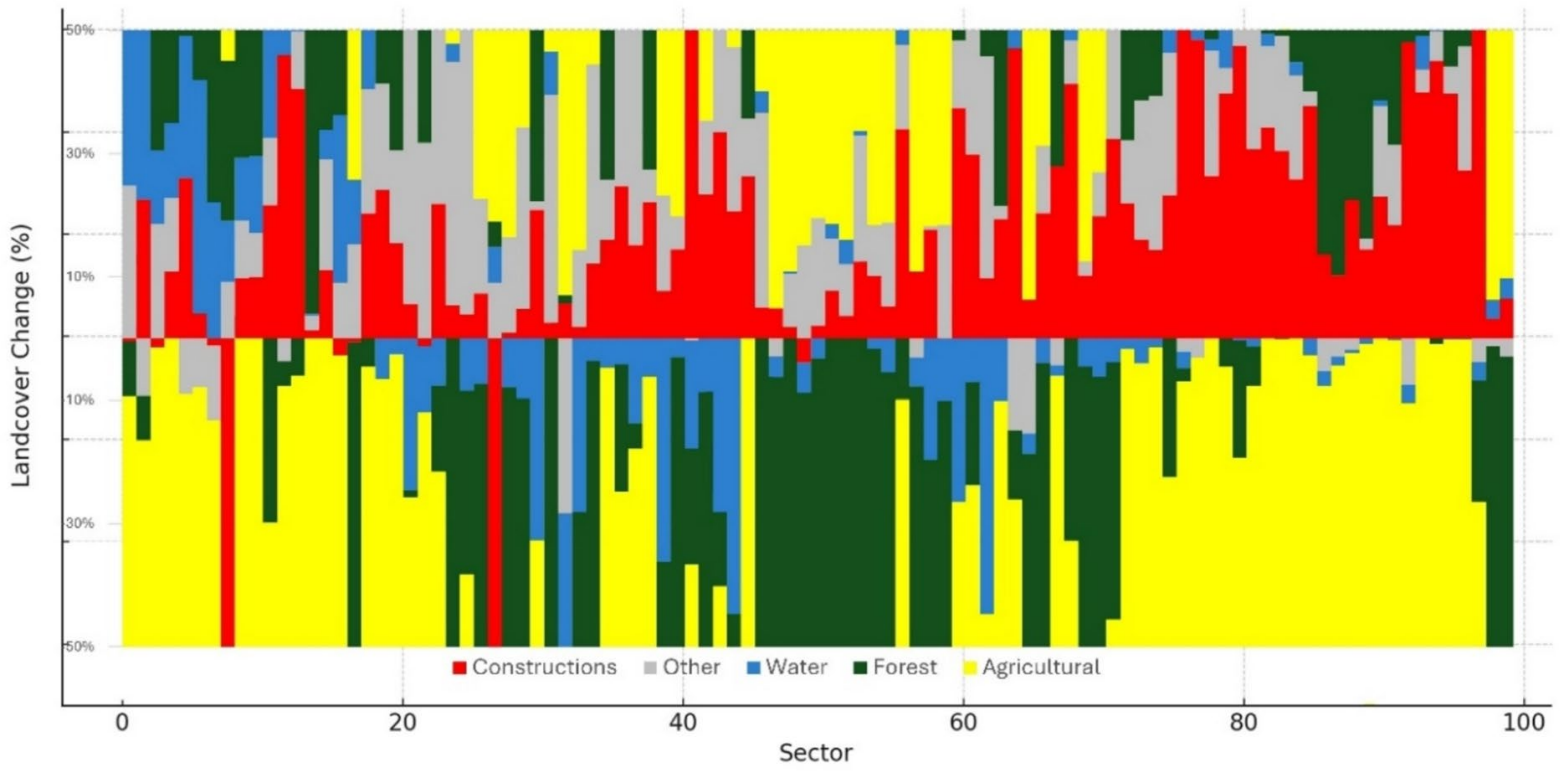
Landcover changes. Each bar represents a sector, with the percentage of change in land cover categories from a baseline year (1900) to the comparison year 2005

The study also observed substantial changes in the land cover within the riparian zone of the Mureș River. When analyzing the land cover changes in various sectors along the Mureș River, several patterns were observed. Overall, we identified considerable changes in land cover along the Mureș River over time, with a notable trend towards increased urbanization, particularly in the higher-numbered sectors, which could correlate with areas closer to urban centers. The variability across sectors emphasizes the complexity of land use changes (Fig. 6).

There is a significant increase in the urban category in almost all sectors, especially towards the higher-numbered sectors. This indicates urban expansion or infrastructure development in those areas. Changes in the “Water” category appear to be both positive and negative across the sectors, suggesting that some areas have seen an increase in water bodies or river width, while others have seen a decrease, which could be due to river engineering or natural changes in the river course. The forest cover has decreased in many sectors, consistent with the increase in urban area, which may have resulted from deforestation for urban development. Agricultural land shows significant decreases in some sectors and increases in others. This could reflect shifts in land use, perhaps from agriculture to urban land cover or reforestation initiatives.

**Fig. 7:**
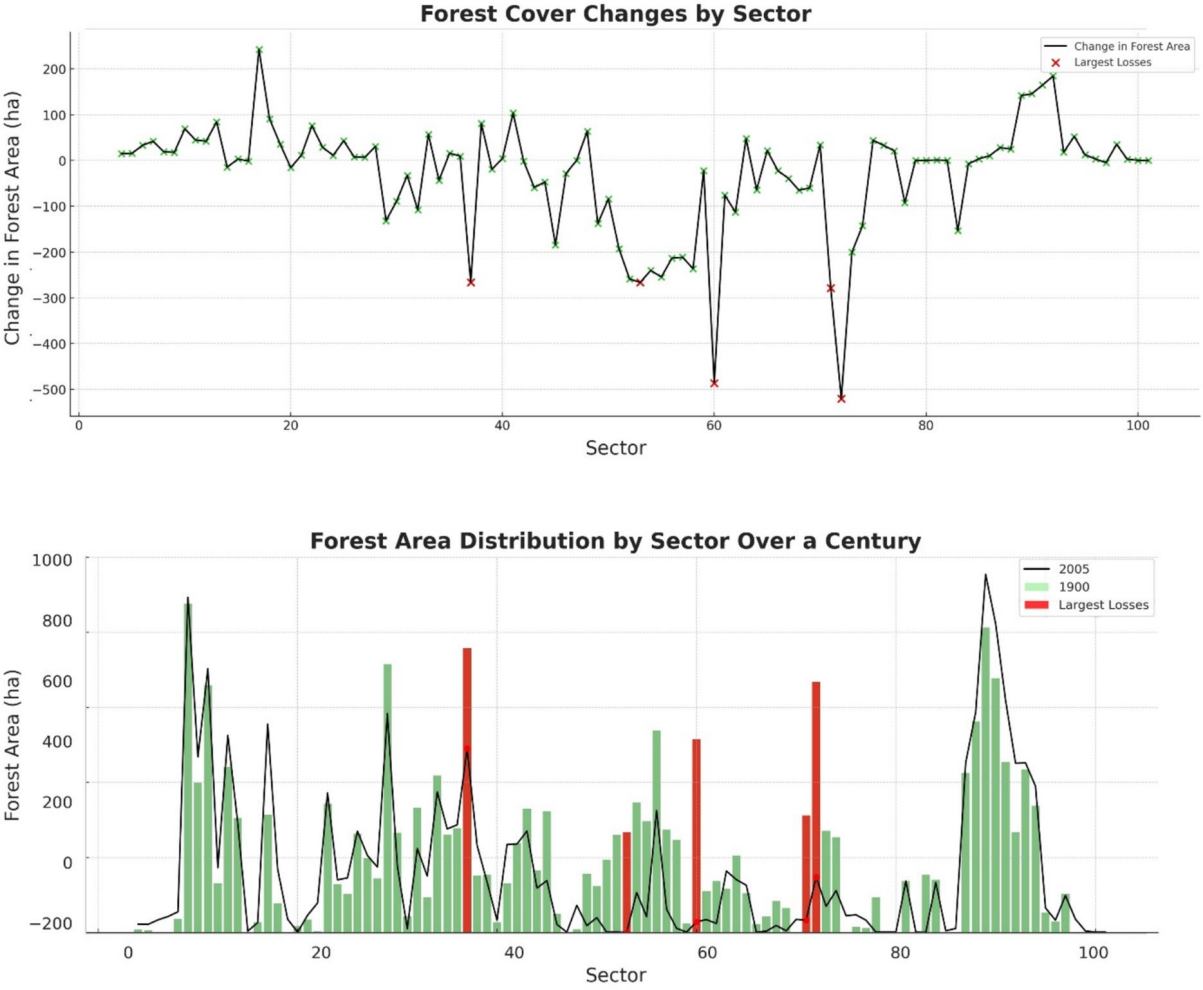
Forest cover changes

Regarding forested areas, there was a notable decrease in forested areas by 16%, with the total area covered by trees reducing from 19,611.12 hectares in 1900 to 16,444.40 hectares in 2005 (Fig. 7).

As for agricultural and urban development, the greatest changes in land cover were observed in sectors 45 to 80, which are predominantly affected by urbanization and agricultural expansion. The area of constructed surfaces increased by 257%, from 5060.37 hectares in 1900 to 13,045.08 hectares in 2005. The loss of riparian vegetation was significant, with a reduction of around 4234.49 hectares over the study period, indicating substantial human impact through land use change.

When compared with the meander changes, urbanization led to channel corrections and management efforts that have potentially influenced the river’s natural meandering process, as suggested by changes in the meandering coefficients in urbanized sectors such as 75 to 87.

### Regression analysis

We performed a regression analysis to explore the relationship between urban area changes (cc) as the dependent variable and changes in forest cover (forest) and water area (water) as independent variables. The results of the regression analysis are summarized in Table 1.

**Table 1:** Ordinary least squares (OLS) regression results

Parameter	Coefficient	Std. error	*t* value	*p* value	95% confidence interval
Intercept	3.2237	0.680	4.739	0.000	[1.873, 4.574]
Forest	− 0.0147	0.026	− 0.562	0.575	[− 0.066, 0.037]
Water	0.5231	2.138	0.245	0.807	[− 3.722, 4.768]

The key metrics of the regression analysis reveal several important aspects of the model’s performance and relationships. The R-squared value is 0.003, indicating that only 0.3% of the variability in urban area changes can be explained by the model. The adjusted R-squared, which adjusts for the number of predictors in the model, is − 0.017, suggesting that the model does not improve when accounting for the number of predictors used. The F-statistic is 0.1596 with a corresponding *p* value of 0.853, indicating that the overall model is not statistically significant. Additionally, the Akaike information criterion (AIC) is 657.9, and the Bayesian information criterion (BIC) is 665.7, which provide measures for model comparison and complexity.

The value of the intercept, which is 3.2237, signifies the expected change in urban area when both forest cover and water area changes are zero, and it is statistically significant with a *p* value less than 0.001. For forest cover, the coefficient is − 0.0147, indicating a slight negative relationship with urban area change; however, this relationship is not statistically significant with a *p* value of 0.575. For river water area, the coefficient is 0.5231, suggesting a positive relationship with urban area change, but this too is not statistically significant, with a *p* value of 0.807. The regression analysis suggests that changes in forest cover and water area do not significantly predict changes in construction within the studied sectors along the Mureș River. The low *R*-squared value indicates that the model explains only a small fraction of the variability in construction changes. This implies that other factors not included in the model might be influencing urban area changes, or the relationship between these variables is more complex than captured by this linear model.

**Fig. 8:**
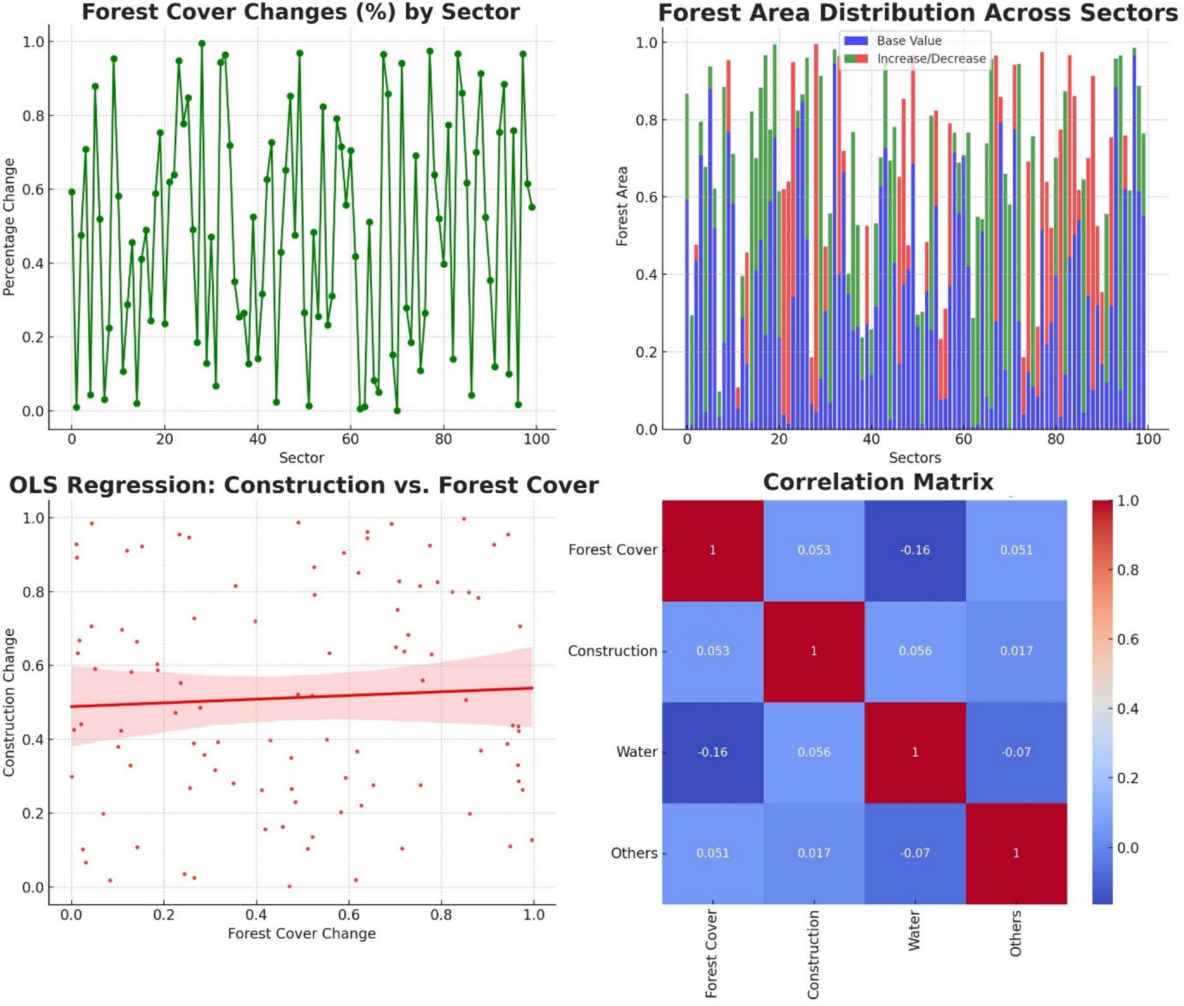
Analysis of land cover changes and relationships in the Mures river riparian zone

The analysis of the relationship between construction (urban area) change and forest cover change is depicted in the scatter plot at the bottom left of Fig. 8. The OLS regression: construction change vs. forest cover change plot shows a slight positive trend between construction change and forest cover change, but the points are widely scattered, indicating a weak correlation. The regression line (in red) suggests a slight increase in construction change with increasing forest cover change, though this relationship is not statistically significant.

Additionally, the correlation matrix in the bottom right of Fig. 8 summarizes the relationships between various land cover types, including forest cover, construction (urban area), water, and others. The low correlation values indicate minimal linear relationships between these variables. For instance, the correlation coefficient between forest cover and construction is 0.053, suggesting a very weak association.

The top panels of Fig. 8 provide further context on forest cover changes. The forest cover changes in percentage by sector (top left) plot illustrates the variability of forest cover changes across different sectors, while the forest area distribution on sectors across one century (top right) shows the base values and increase/decrease trends in forest area across sectors over the study period.

These plots collectively indicate that the relationships between construction change and forest cover, as well as other land cover variables, are complex and not well-explained by simple linear correlations. This highlights the need for further investigation into the potential non-linear or multi-factor interactions affecting land cover changes in the Mureș River riparian zone.

## Discussion

This study represents an extensive analysis of the morphological changes in the Mureș River channel and the accompanying land cover changes within the riparian zone over a century, from 1900 to 2005. By employing advanced geomatic techniques, we have uncovered significant patterns of change that illuminate the river’s response to both natural dynamics and anthropogenic pressures. The integration of historical cartographic data with contemporary GIS tools has enhanced our ability to analyze long-term changes with greater accuracy and detail than was previously possible.

### River channel geometry changes

The observed decrease of approximately 7.6% in the river’s surface area signifies substantial geomorphological transformations. This reduction is indicative of processes such as sediment deposition, channel incision, and bank stabilization efforts. The variability of changes across different sectors underscores the complex interplay of natural processes and human activities influencing river morphology.

In sectors like 42 and 69, the significant increases in meander length (49% and 60%, respectively) suggest enhanced lateral erosion and sediment transport dynamics. These changes may be attributed to natural adjustments to altered hydrological regimes or increased sediment loads resulting from upstream land use changes. Similar phenomena have been documented in other river systems, such as the Mississippi River (Hudson & Kesel, 2000) and the River Meuse (Makaske et al., 2009), where increased meandering was linked to both climatic factors and land use alterations.

Conversely, sectors 43 and 44 exhibited substantial decreases in meander length (30% and 50%), indicating channel straightening. Such modifications are often associated with river engineering interventions like channelization and bank reinforcement aimed at flood control, navigation improvement, or land reclamation (Campbell et al., 2010). These human-induced changes can disrupt the river’s natural equilibrium, leading to altered flow velocities, reduced sediment deposition in floodplains, and increased downstream flood risks (Wohl, 2019).

The alterations in meander amplitude and sinuosity further reflect the river’s morphological adjustments. Increased meander amplitudes in some sectors point to active meander development and potential instability in channel positions (Yarnell & Thoms, 2022). In contrast, reduced amplitudes and sinuosity in other areas may result from constraints imposed by infrastructure development or deliberate river training works.

### Riparian zone land cover changes

The land cover analysis within the riparian zone revealed substantial anthropogenic impacts. The 257% increase in urban development is a clear indicator of significant human encroachment into the riparian areas. Urban expansion often leads to the construction of impermeable surfaces, which can alter hydrological responses by increasing surface runoff, reducing infiltration, and exacerbating peak discharge rates during storm events (Walsh et al., 2012). These changes can amplify the erosive power of the river, potentially affecting channel morphology.

The 16% reduction in forested areas, amounting to a loss of 4234.49 hectares, raises concerns regarding ecological integrity and river health. Forests in riparian zones play a critical role in stabilizing banks, filtering pollutants, regulating microclimates, and providing habitats for diverse species (Ali & Mahrukh, 2023). Deforestation can lead to increased bank erosion, higher sediment loads, and degradation of aquatic habitats (Luke et al., 2007). The loss of riparian vegetation observed in the Mureș River basin mirrors global trends where riparian zones are under pressure from agricultural expansion and urban development (Ali & Mahrukh, 2023; Jurcoane et al., 2023).

### Relationship between morphological changes and land cover transformations

Despite the significant changes observed in both river morphology and land cover, the statistical analyses did not reveal a significant linear correlation between them. The low *R*-squared values in the regression models suggest that the relationship between land cover changes and river morphology is complex and possibly influenced by additional factors not accounted for in the models.

One possible explanation is the influence of hydrological alterations resulting from upstream dam construction, water abstraction, or climate variability, which can affect flow regimes independently of local land cover changes (Aguiar et al., 2016). Additionally, direct river modifications such as channel dredging, embankment construction, and other engineering works can alter morphology regardless of adjacent land use (Poff et al., 2007).

### Accuracy, limitations, and the role of geomatic techniques

The use of historical maps and modern GIS tools allowed for a detailed multi-temporal analysis; however, certain limitations must be acknowledged. The accuracy of historical cartographic data can be affected by the surveying techniques and cartographic standards of the time, potentially introducing errors in georeferencing and measurements. Moreover, the classification of historical land cover relied on map symbology and annotations, which may not capture all nuances of land use.

The classification accuracy for contemporary data was enhanced through ground truthing and accuracy assessments, achieving overall accuracies above 85%. Nevertheless, misclassifications can occur due to spectral similarities between different land cover types or changes in classification schemes over time.

Despite these limitations, the integration of geomatic techniques has significantly improved our ability to monitor and analyze morphological evolution and land cover changes. The combination of high-resolution spatial data, advanced analytical tools, and historical records provides a robust framework for understanding long-term environmental changes (Nita et al., 2018). Such methodologies are invaluable for detecting subtle changes, identifying trends, and informing management decisions.

### Anthropogenic impacts and management implications

The findings highlight the profound impact of human activities on the Mureș River’s morphology and riparian environment. Urban development, deforestation, and agricultural practices have altered the natural processes governing river dynamics. These anthropogenic pressures can lead to reduced resilience of river systems, making them more susceptible to environmental changes and extreme events (Hudson & Kesel, 2000; Naiman & Décamps, 1997).

From a management perspective, the study underscores the necessity for integrated river basin management approaches that consider both geomorphological processes and land use practices. Strategies should aim to balance socio-economic development with ecological conservation. For instance, implementing riparian buffer zones can help mitigate the impacts of land use changes by providing space for natural river adjustments and reducing pollutant loads entering the river (Naiman & Décamps, 1997; Namour et al., 2015).

Reforestation and afforestation efforts in the riparian zone can enhance bank stability, reduce erosion, and improve habitat connectivity (Mondal et al., 2018). Sustainable urban planning that incorporates green infrastructure can mitigate the adverse effects of urbanization on hydrological regimes (Walsh et al., 2012). Additionally, restoring natural meandering patterns through river rehabilitation projects can improve ecological function and resilience (Kondolf, 2006).

## Conclusion

This study has successfully quantified the morphological changes of the Mureș River channel and the shifts in land cover within its riparian zone over a century. The significant decrease in river surface area, alterations in meander characteristics, substantial increase in urban development, and reduction in forested areas reflect the combined influence of natural dynamics and human activities. The lack of significant correlation between land cover changes and river morphology suggests that other factors, such as hydrological alterations and direct river modifications, play crucial roles in shaping the river’s evolution.

The findings emphasize the importance of considering both geomorphological processes and land use practices in river management and conservation strategies. An integrated approach is essential to address the complex interactions affecting river systems. Continued monitoring using advanced geomatic techniques is vital for informing adaptive management and ensuring the sustainability of river ecosystems.

Future actions should focus on implementing integrated management strategies that promote ecological conservation while accommodating sustainable development. This includes reforestation initiatives, sustainable urban planning, restoration of natural river dynamics, and the establishment of policies that mitigate the impacts of anthropogenic activities on river morphology and riparian environments. By fostering a balance between development and environmental stewardship, we can enhance the resilience of river systems like the Mureș River, ensuring their ecological integrity and the services they provide for future generations.

## Data Availability

No datasets were generated or analysed during the current study.
